# Gynæcological Medicine

**Published:** 1871-07

**Authors:** T. S. Powell, W. T. Goldsmith


THE GEORGIA

MEDICAL COMPANION:

A MONTHLY ADVISER.

VOL. I. ATLANTA, GA., JULY, 1871. NO. 7.

### PART I.

### Original Communications and Special Selections.

### GYNÆCOLOGICAL MEDICINE.

*Report of the Committee of the Seventh Congressional District of Georgia, upon "Gynæcological Medicine," prepared to be read before the Georgia Medical Association. By T. S. Powell, M. D., and W. T. Goldsmith, M. D.*

[CONTINUED FROM LAST NUMBER.]

Our tent being thus prepared, it is introduced into the canal, where it acts by its gradually distending pressure, exerting a wholesome stimulating effect upon the diseased surfaces, particularly if granular degeneration has occurred, and affording easy egress for the discharge of the secretions. After the removal of the tent, which should not be allowed to remain longer than 24 hours, the canal should be thoroughly cleansed of mucus and blood, which should be cautiously and gently accomplished by saturating in water small portions of cotton rolled around a piece of whalebone in such a manner as to preserve the long axis of the rolled cotton to the staff used. After this, a small, slender syringe may be employed to wash out the canal—carefully avoiding its introduction into the uterine cavity. Unless the canal is thoroughly cleansed, our applications will not only be rendered inert by being neutralized by the mucus secretions, but we will fail to give the stimulation we desire to the diseased surfaces. Gynæcologists differ widely as to what substances best meet the indications for the purpose, when applied, as well as to the strength with which they should be used. Nitrate of silver, acid nitrate of mercury, iodine, sulphate of copper, chromic acid, carbolic acid, and various astringents as well as bismuth and styptic colloid, have been employed. We usually employ the nitrate of silver, either in the solid form or in solution. In most cases we have found this sufficient. There occur cases, however, where an eschorotic is demanded. Dr. Bennett recommends potassæ cum calce, protecting the parts by ascetic acid for this purpose, while Dr. Marion Sims employs chromic acid. The latter we prefer, as its application does not result in cicitrization and contractions of the canal, which will frequently be induced by the former. Indeed, nitrate of silver is not altogether free from causing this result, if improperly used. Chromic acid may be employed in its full strength, or weakened to any desired strength by dilution. Whatever medication is used, it should be painted over the entire surface of the canal—from the os internum to the reflected vaginal mucous membrane. These applications may be repeated every five or eight days, according to the amount of stimulation produced, and should be preceded each time by the introduction of the tent twenty four hours before being employed. Applications may be made to the cervical canal, with or without dilitation, by using the "uterine probe" and "applicator"—two instruments originated by Dr. T. Emmett, of New York. The probe is a small ductile silver sound, taking any curve given it. This is used to pilot the way for the "applicator," which is moulded to the same curve, and carries the fluid soaked in cotton twisted on it. These instruments are, however, more particularly designed for treating chronic endometritis, with or without flexure. In making applications to the cervical canal, the os internum should never be passed—when applied either by the syringe or instruments. In using Dr. Emmett's probe, with slide, cotton should be wrapped nicely upon its extremity, when being dipped in solution of nitrate of silver, Churchill's tr. iodine, or chromic acid may be carried to the part, and if desired, may be left in the canal by being detached by the slide. Two applications, for one or two minutes, are generally necessary.

In applying the solid nitrate of silver, the usual means for this purpose are inefficient. The common port-caustic, will not carry the stick far enough. Many instruments have been suggested to overcome this difficulty. We, however, know of no instrument so well adapted to the purpose as the probe of Prof. F. D. Lente, of New York city, and manufactured by Geo. F. Tieman & Co.

It is similar to the uterine probe, having a rounded extremity and a bulbous swelling a short distance from its uterine extremity.

The rounded end of the instrument is dipped frequently and rapidly cooled, in melted nitrate of silver, which has been fused in a little platnum cup by a spirit lamp. Having thoroughly prepared the canal by dilitation and cleansing, this is carried to every part of the cervical surface, after which the os should be well syringed, and a pellet of cotton, saturated with glycerine, applied to it, first attaching a string for its removal.

The use of medicated tents in treating chronic cervical endometretis, will be fouud highly beneficial. Dr. J. Gaillard Thomas prefers it now to any other mode. The carbolized tent will largely accomplish this purpose. If, however, nitrate of silver, iodine, lead, sulphate of copper or iron be preferred, they may, by being incorporated with cocoa-butter, be introduced into the canal of the tent left by the holder. The heat of the parts will melt the butter, and the medication be carried to every part of the cervical canal; or the sponge, before being formed into tents, may be soaked in various medicated solutions.

We will next notice *chronic corporeal endometritis* as naturally following that of cervical endometritis, being an extension, in many instances, of the latter disease, and involving similar structures. The pathological changes occurring in the lining membrane of the uterus is now receiving from gynæcologists much attention, which condition consists in thickening, increased vascularity, and hyper-secretions, from the numerous glands studding the lining membrane, which, under various names, is known as uterine catarrh, uterine leucorrhœa, etc. When the uterus is examined after death occurring from endometritis or during its progress from other complaints or causes, its cavity is found enlarged, with its walls thinner than normal, and most frequently displaced by some form of flexure. The lining membrane is generally more or less deprived of its epithelium; is red; congested in places, and rough by the enlargement of its blood-vessels, and distended mouths of the utricular follicles. These follicles are, doubtless, the seat of the disease: and the hyper-secretions from which, in all cases, constitutes the uterine leucorrhœa, the most prominent symptom of the disorder. Chronic corporeal endometritis may very properly be divided into two forms: First—Where the middle and lower portions of the cavity is implicated; and secondly, where the fundus is the seat of the inflammatory action.

Dr. Routh thinks that fundal endometritis is a disease of great gravity, and explains the radiations of pain by referring to the anatomical arrangement of the nerves of the uterus, the fundus being supplied by a distinct set of nerves, in direct relation with the semiluna ganglia. He divides fundal endometritis into four varieties:

1. The convulsive form, in which the fundal pain, passing down one of the thighs, increased by passing the sound, &c., was accompanied by a variety of convulsive seizures, such as spasmodic vomitings, tetanus, hysterical fits, with or without mania, up to epileptoid fits and catalepsy; the symptoms persisting for some time until the catamenial function was fully established, or flooding occurred. 2. Inflammation of the fundal membrane with increased secretion, accumulation of this in the cavity from obstruction at the inner os, giving rise to symptoms of pregnancy or even labor, with intense fundal pain, all relieved by a sudden gush of discharge, persisting for months, and often recurring. 3. Chronic cases of Gooch's irritable uterus, with more or less complete loss of the power of walking. Here also the inflammation of the fundus was unmistakable. 4. Cases of acute fundal endometritis which sometimes rapidly passed into metro-peritonitis, generally fatal, although sometimes assuming a more chronic type.

Mr. Gaskoin was particularly struck with that part of Dr. Routh's paper which referred to coxalgia as a symptom of endometritis.

The causes of chronic corporeal endometritis are similar in many respects to those inducing cervical endometritis, such as exposure to cold during menstruation, with sudden arrest of the flow; sexual intercourse, etc. There are, however, causes which induce the disease from the peculiar construction of the uterus itself, and the function which it is its office to perform. The menstrual blood, by obstructions in the os internum, often becomes arrested, in the cavity, clots by admixture with its alkaline secretions—thus irritating and inflaming the lining membrane—or, it may result from placental remains after abortion or parturition; or by extension of cervical endometritis; from mechanical injuries, such as efforts made by instruments in producing abortion, or to tumors in the uterine walls. It sometimes occurs as a complication with exanthemetous diseases. The symptoms of chronic corporeal endometritis are so similar to those of cervical endometritis, as to often greatly perplex the gynæcologist in arriving at a correct diagnosis. They both have, in common, leucorrhoœa, menstrual disorders; bearing down sensations in the pelvic cavity; nervous disorders; general debility; pain in the back, etc. The pain, however, usually attending corporeal endometritis, is felt midway between the sacrum and pubes i. e. in the uterus. There is often tympanitis and conditions closely simulating pregnancy, while sterility is so generally present as to form one of the characteristics of the disease. The leucorrhœal discharge, if free from admixture with that of the cervical canal, will often afford evidence of its uterine origin. As before remarked, the discharge from the follicles of the canal, under diseased action, is tenacious, glairy and thick—while that flowing from the utricular follicles of the uterine cavity, is not so thick, glairy or tenacious; and is frequently mixed with blood assuming a "rust-colored" appearance, which, Dr. Bennet regarded "as characteristic of internal metritis as the rust-colored expectoration is of pneumonia." Dysmenorrhœa is often a symptom; the membrane, during the pain, being exfoliated at the menstrual period, and should be attributed to endometritis. Hysterical symptoms usually occur—neuralgia of the head, with melancholly forebodings and mental incapacity.

According to our experience, corporeal endometritis is a rare disease compared with cervical endometritis. The discrepancies of authors on this point is great. Drs. Bennett, Byford, and Gaillard Thomas, believe it a disease of not frequent occurrence. Prof. Thomas remarks that "the most frequent locality of uterine inflammation is that portion of the uterus below a line running across it, through the os internum." This corroborates our experience, of uterine diseases. He thinks, however, that that portion of the organ above this line is of more frequent occurrance than supposed by Dr. Bennett. On the other hand, Drs. West, Tilt, and Aram, believe endometritis to be a common disease. No doubt, greater facilities for arriving at correct diagnosis under the impulse of modern investigation will prove the opinion of Prof. Thomas to be the true one; at least, so far as our experience goes it coroborates his view of the comparative frequence of these diseases.

Many of the physical signs relied upon as indicative of corporeal endometritis are vague and indefinite. The most indubitable evidence of its existence is the absence of cervical endometritis—a leucorrhœal discharge being present. By passing the finger of one hand in the rectum and placing the other upon the abdomen above the pubis, and then approximating the finger thus introduced and the hand on the abdomen, the uterus may frequently be caused to reveal a certain amount of pain. Dr. Sims' uterine probe may be carried into the uterus cavity and suddenly its extremity struck against the lining membrane. If endometritis is present, pain, sometimes intense, will be complained of by the patient.

The prognosis of chronic corporeal endometritis in its worse form is not hopeful, under any treatment. There are, however, many cases amenable to proper medication. In patients where the constitution is unimpaired; when the system is not seriously involved; when the patient is nearer the "term of life," or when the case is of recent origin. If the menstruation is not seriously complicated with dysmenorrhœa, in which the membrane is thrown off in shreds; if the leucorrhœal discharges contains no pus, if inflammation of its parenchyma has not occurred, inducing displacements, then the prognosis is more favorable than when the opposite condition exist. The serious nature of corporeal endometritis arises from its complications with other diseases. The inflammation may extend to the peritoneum through the fallopian tubes, lighting up pelvic peritonitis, or the displacements caused by complication with corporeal metritis may induce cystitis, rectitis and cellulitis, and frequently ovaritis. The discharges, when purulent, often cause vaginitis.

The general principles recommended in the treatment of chronic cervical endometritis should be enforced. Our experience in these diseases is, that without due attention to these general principles, no treatment, however excellent in other respects, will avail much in its subjugation. Fresh air, good diet, regular habits, with moderate exercise, and quiettude of the mental and emotional nature, should be insisted upon. The digestive functions require special notice and attention. All stimulants, and condiments, as aromatics and spices, should be prohibited. Change of air and water will often be beneficial. The pelvic cavity should be freed from pressure, and all employments causing strain or friction upon the uterine organs should be avoided; as lacing, lifting weights, or working sewing machines.

The direct application of remedial agents in the treatment of corporeal endometritis has employed the consideration and attention of the most zealous gynæcologists of the day. Prof. E. R. Peaslee places intra-uterine medication under two divisions—endometrical injection and ingestion. The subject of intra-uterine injections has of late received much attention and became a fruitful source of discussion. Vidal de Cass s asserts that fluids thrown into the uterine cavity will not pass through the fallopian tubes into the abdomenal cavity, if not made with excessive force, while Prof. Thomas thinks this is liable to happen. Others believe evil results due to too large quantity of fluid used; to the force with which they are injected; to the peculiar re-action of the organ against all foreign bodies; to the enterance of the injection into the open vessels of the mucous membrane, and sometimes to a purely hysterical condition. The weight of testimony is decidedly adverse to the employment of intra-uterine injections. Dr. Peaslee asserts, however, that by attention to certain preliminary rules, intra-uterine injections may be performed without the least danger. The verdict, however, of the best gynæcologist is against it. Prof. Thomas thinks it does not constitute and advance in the treatment of endometritis. Prof. Peaslee asserts that "all bad effects from injection may be guarded against by adopting these precautions:

1. "Let the water be blood-heat or a little less.

2. Introduce it slowly and carefully so as not to disturb the uterine cavity. Only ten or fifteen drops are needed to fill this in a virgin, and twenty-five to forty in one who has borne children.

3. Be sure to provide for a return of the overplus of water by the side of the instrument. This implies a previous dilatation of the cervical canal to a diameter greater than the tube of the syringe.

4. Abstain from the use of all injections, if on introduction of the sound into the uterine cavity, there is found to be tenderness near the fundus, for, in such a condition, even the contact of warm water, will often cause intense agony. If there is no special sensibility of the endometrium, injections, with the precautions enumerated, may be regarded as safe."

He thinks that the method of intra-uterine medication by ingestion as usually practiced, by being applied over the surface as paint, has no advantages over injections; but when applied in the proper condition, by proper means, it is not only perfectly safe, but more efficacious than injections. If say, 15 or 20 drops of the liquid used, be applied by means of cotton, there would never be any injury done. The solid nitrate of silver, in some instances has proven an unsafe remedy. He thinks that ointments have no advantages over fluids, and may be displaced by them entirely in practice.

"Endometrical ingestion is required in few of the cases the gynæcologist has to treat; while injection should far more seldom be resorted to, and never unless preliminary to ingestion of curative agents, in cases where there is no other way to remove blood or secretion, or in cases of metrorrhagia, where styptics are required. For cleansing the endometrium, a solution of salt is most valuable, and if there is no special sensitivenass a very little soap may be added. For medicaments, the tincture of iodine is most valuable in metrorrhœa, being used at first ʒ ss to ℥ i of water, then ʒ i to ℥ i, and so on, up to the full strength, if needed.

The sulphate of zinc may be used grs. v. to ʒ i of water at first, and then the nitrate of silver grs. v. to ʒ i to the ℥ i of water; the tannic acid from ℈ i to ʒ i to ℥ i of glycerine, and chloride of zinc ʒ ss to ℥ i of glycerine.

If chromic acid is used, it should be with great caution, and not over one part to ten of water used at first.

In metrorrhagia, the per sulphate or per chloride of iron, bears the palm.

To dilate the cervical canal preparatory to injection or ingestion, the sponge tent is the proper method if the cervix is firm and indurated. If the cervix is, however, lax and easy of dilatation, Prof. P. has found very satisfactory a set of steel dilators of his own devising.

There are five dilators in the set, from one-tenth to five-sixteenths of an inch in diameter. Each has a bulb one and three-quarter inches from its point, so that it can pass only that distance into the uterus, and therefore projects less than an inch into its proper cavity, The instrument may be passed without the use of the speculum, and often the full dilatation may be accomplished at one visit. The dilatation should be carried to at least three-eighths of an inch, whether the fluid is to be injected or ingested.

To accomplish the reflux of fluid in injection, the Professor has devised a tube with conical extremity, three-eighths of an inch in diameter, and two inches long. On the sides of the cone are three fenestra, half an inch long each; there is also a fine opening at the end of the tube. When this is inserted the fenestra open into the cavity of the uterus. The fluid is injected through one of the fenestra by means of a syringe with a nozzle perforated at its sides, but not extremity; it flows out readily through the fenestrated openings.

In intra-uterine ingestion, in order to be sure of carrying the remedy and applying it directly to the diseased surface, Professor Peaslee has devised a tube one and three quarter inches long, and with only two fenestræ. This tube being passed into the uterine cavity, the medicament is carried through the fenestræ and painted over the whole of the endometrium at will, by a mass of cotton."

Dr. C. D. Palmer uses "a silver tube, (No. 7) 8½ inches long, which is attached by a screw to a perfectly accurately working syringe, like Anel's, of the capacity of half an ounce. This tube, with four instead of six bars, to permit greater freedom in the exit of fluid, has an appropriate curve, for easy introduction. The bars extend four inches from the distal extremity, to suit the length of various uteri. The cap has nine minute perforations; one at the extremity of the tube, and two on each face. This canula can usually be introduced into most uteri without difficulty, through, or without the use of, the speculum. Occasionally previous dilation is required, for which purpose Kammerer's metallic bougies may be used. The expense, time, trouble, not to say pain and danger, of sponge tents, are entirely unnecessary. The half ounce of fluid is slowly and gently injected; the greater portion of which, by the cap arrangement, is rapidly thrown back, together with whatever of secretion may have been retained within the uterus. The fundus is washed off by the current through the perforations. Retention of fluid within, and consequent distention of, the uterine cavity are impossibilities. No air is mixed with the fluid because of the accurate working of the piston; the force of stream and the quantity of fluid are regulated *ad libitum.* If necessary, the syringe can be unscrewed at its junction with the tube, refilled with fluid, or medicine, and injected without withdrawal of the latter."

Dr. Palmer thinks that the practice of intra-uterine injections, although disapproved of by so excellent teachers and practitioners as Thomrs, Storer and others, is gaining favor among gynæcologists."

"Using this fluid I have repeatedly injected the uterus of a considerable number of patients without occasioning any more pain than is produced by the introduction of any ordinary sound, and have also used medicated fluid with but little more pain than results from the application of the same agents by means of the applicator. Now, the injection of salt water alone, in many cases of uterine catarrh, proves highly curative, and will often be found of itself sufficient. If not, after washing out all secretions, the application of the necessary agent may be made with the probe and cotton. Such a procedure, in the majority of instances, is preferable, and more advisable than injection of the same agent, being safer, and after the preliminary cleansing of the surface, sufficiently thorough. There are two agents in particular in the treatment of uterine catarrh, which may be employed with this canula advantageously, and in certain cases, when there is due tolerance of the organ, safely. These are diluted Churchill's tincture of iodine, and diluted carbolic acid, first in weak solution, and then of gradually increased strength.

On the contrary, Dr. Palmer asserts that "sponge tents have done much harm in some hands. We feel confident that their frequent use, as often now recommended, is not as free from danger as some would lead us to suppose. Peaslee well puts it when he says, "Applications to the endometrium require a delicate surgical dexterity, and those who possess neither tact nor experience in this direction will probably do more mischief than benefit."

In making application to the uterine cavity, a proper speculum should be used. The common cylindrical speculum is not only inconvenient, but attempts to reach the cavity through it, are attended with danger. Sims' speculum, of all others, is decidedly the best; but this instrument requires a third person as assistant, and to be thoroughly understood in its adjustment to the proper position of the patient. A modification of Sims' speculum, with depressor attached, the "telescopic speculum" of Prof. Thomas, or Prof. Storer's improved Cusco's speculum should be preferred in private practice. In making applications by means of cotton, we find the uterine probe of Dr. Sims of great value, being better than Simpson's uterine sound for exploring the uterine cavity, from the fact that it is smaller and easily adapts itself to the direction of the canal, by its flexibility. The best method of making these applications, is the one employed by Dr. Sims. He first introduced the uterine probe to determine the extent and direction of the cervical canal and uterial cavity. It should ever be borne in mind that mal-position of the uterus is a common attendant upon corporeal endometritis—metritis being often one of its complications. Having ascertained the direction, first cleansing the cervical canal, then withdraw the probe, place the dressing-probe by its side, giving the latter the same curve, wrapping the end with a small flock of cotton, he passes it up to the fundus cleansing gently, every portion of the surface of the canal and cavity. Withdraw this—another piece of cotton is wrapped as at first, after being dipped in the fluid to be used, is again carried to the fundus, where he keeps it stationary from half a minute to a minute. He makes only one application. After this treatment, the patient should be kept quietly in bed for several days. The strength of the solutions have been already given.

Prof. Thomas knows of no method equal to that of medicated tents. Of these we have already spoken. He advises that they should be passed completely up to the fundus, and withdrawn in 24 hours, by a thread attached. They should be employed once a week. Not the least advantage arising from their use, is the pressure they exert upon the diseased lining membrane.

"Most cases of corporeal endometritis, and of general endometritis, if of any duration, are attended with marked enlargement of the uterine cavity. Such a condition of things is exceedingly unfavorable in a number of ways. Large quantities of blood, mucus and pus here accumulate, which we never succeed in completely removing by the ordinary methods. The use of the canula in such cases is attended with special advantage. The fluid used for cleansing purposes is salt water, from v. to i. grs. of common salt to ℥ i. of clear water, at the temperature of 98 deg. F.

*Chronic cervical metritis.*—This disease is a very common complication of cervical endometritis, and involves the parenchyma of the cervix. It is generally the result of abortion or parturition. It is caused, however, by injuries of the cervix, and frequently by excessive coition. For these reasons it is rare in women who are unmarried or have not borne children. The finger feels an enlarged cervix—it is lower than normal; the os is usually patulous and painful to pressure. It is one of the causes of prolapsus, and frequently of other displacements. Sometimes the cervix is extremely hypertrophied. We have seen it quite two inches in diameter. This disease takes its starting-point from congestion —advancing to inflammation, effusion occurs. In this condition it may remain for a considerable length of time—sometimes suppuration takes place, or the cervix becoming smaller, remains indurated. The treatment of chronic cervical metritis should be governed by the condition of the system, the part receiving the necessary attention in order to remove chronic inflammatory condition of the part. The general treatment should be influenced by measures to restore health, by good, nutritious diet. If the cervix is painful, and presents evidences of engorgement, we usually apply leeches, or scarify the parts. Sexual intercourse should be prohibited, and the patient kept at rest. In leeching the cervix, it should be remembered that should they fasten upon the lips of the os, or within the canal, they produce intense pain. In order to prevent this, a pellet of cotton, as recommended by Dr. Bennett, should be placed within the os, having a string attached for its removal. A few punctures should be then made at several points, and the leeches placed within the speculum, a small quantity of cotton covering them down to the cervix. This process should be watched throughout, and as soon as they fall off, the clots should be removed carefully, before withdrawing the instrument. Vaginal injections should be used as recommended for ulceration of the os uteri ; and medicated pesseries and suppositories will be found useful, especially if anodynes are called for by pain. If the disease resists the local blood-letting and emollient applications, we resort to counter irritation to destroy, at certain points, the epithelium. The vessicating cullodion or the cauterizing irons, may be used for this purpose. We prefer, however, the solid nitrate of silver, which, when applied to the vaginal cervix, destroys the epithelium and, becoming detached, leaves a bare surface. In obstinate cases the potassæ cum calce may be used, protecting the os and vaginal surfaces by cotton saturated in ascetic acid, squeezed dry, and then the parts covered with it, as recommended by Dr. Bennett.

When the hypertrophied cervix resist all mild forms of treatment, the actual cautery will be found of value, especially if it be complicated with corporeal metritis and inveterate ulceration and growths of a malignant character. The application of the cautery should be made cautiously, and the iron held to the parts, brought to a white heat, some distance from the os, for a few seconds only, a horn, ivory or wood speculum, being used. Several points of the cervix should be thus cauterized. A copious injection of tepid water is then to be thrown upon the cervix, and a pellet of cotton, saturated with glycerine, applied before the speculum is withdrawn. These injections should be frequently repeated, and the patient kept quietly in bed. When the eschar falls off, the exposed surfaces should be stimulated by applications of nitrate of silver, tr. iodine, etc., followed by emollient applications.

